# Fitness of *Leishmania donovani* Parasites Resistant to Drug Combinations

**DOI:** 10.1371/journal.pntd.0003704

**Published:** 2015-04-07

**Authors:** Raquel García-Hernández, Verónica Gómez-Pérez, Santiago Castanys, Francisco Gamarro

**Affiliations:** Instituto de Parasitología y Biomedicina “López-Neyra”, IPBLN-CSIC, Parque Tecnológico de Ciencias de la Salud, Granada, Spain; University of Antwerp, BELGIUM

## Abstract

Drug resistance represents one of the main problems for the use of chemotherapy to treat leishmaniasis. Additionally, it could provide some advantages to *Leishmania* parasites, such as a higher capacity to survive in stress conditions. In this work, in mixed populations of *Leishmania donovani* parasites, we have analyzed whether experimentally resistant lines to one or two combined anti-leishmanial drugs better support the stress conditions than a susceptible line expressing luciferase (Luc line). In the absence of stress, none of the *Leishmania* lines showed growth advantage relative to the other when mixed at a 1:1 parasite ratio. However, when promastigotes from resistant lines and the Luc line were mixed and exposed to different stresses, we observed that the resistant lines are more tolerant of different stress conditions: nutrient starvation and heat shock-pH stress. Further to this, we observed that intracellular amastigotes from resistant lines present a higher capacity to survive inside the macrophages than those of the control line. These results suggest that resistant parasites acquire an overall fitness increase and that resistance to drug combinations presents significant differences in their fitness capacity versus single-drug resistant parasites, particularly in intracellular amastigotes. These results contribute to the assessment of the possible impact of drug resistance on leishmaniasis control programs.

## Author Summary

Chemotherapy is currently the only treatment option for leishmaniasis, a neglected tropical disease produced by the protozoan parasite *Leishmania*. However, first-line drugs have different types of limitations including toxicity, price, efficacy and mainly emerging resistance. The WHO has recently recommended a combined therapy in order to extend the life expectancy of these compounds. The emergence and spread of *Leishmania* antimonial-resistant parasites have led to a high rate of antimonial failure in India and have raised questions about the selection and propagation risk of drug resistant parasites. The spread of drug-resistant parasites in the field probably depends on their transmission potential, which is influenced by, among other factors, the relative fitness of drug-resistant versus drug-susceptible parasites. In light of this, we have designed experimental studies to determine whether *Leishmania donovani* parasites resistant to single and combinations of anti-leishmanial drugs present any advantages in their ability to bear the different stress conditions versus a susceptible *L*. *donovani* line. Our results suggest that resistant parasites acquire an overall fitness increase and that resistance to drug combinations presents significant differences in their fitness capacity, particularly in intracellular amastigotes.

## Introduction

Leishmaniasis, a neglected tropical parasitic disease that is prevalent in 98 countries spread across three continents, is caused by protozoan parasites belonging to the genus *Leishmania* [1]; visceral leishmaniasis (VL), caused by species of the *Leishmania donovani* complex, is a lethal disease if left untreated. The recommended first-line therapies for VL include: i) pentavalent antimonials (meglumine antimoniate and sodium stibogluconate), except in some regions in the Indian subcontinent where there are significant areas with drug resistance [2]; ii) the polyene antibiotic amphotericin B or the liposomal amphotericin B formulation AmBisome; iii) the aminoglycoside paromomycin; and iv) the oral drug miltefosine. Although WHO [1, 3] recommended the use of either a single dose of AmBisome or combinations of anti-leishmanial drugs in order to reduce the duration and toxicity of treatment, to prolong the therapeutic life-span of existing drugs and to delay the emergence of resistance, recent experimental findings have demonstrated the ability of *Leishmania* to develop experimental resistance to different drug combinations [4]. The emergence and spread of *Leishmania* antimonial-resistant parasites have led to a high rate of antimonial failure in India [5] and have raised questions about the selection and propagation risk of drug resistant parasites [6, 7]. The spread of drug-resistant parasites in the field probably depends on their transmission potential, which is influenced by, among other factors, the relative fitness of drug-resistant versus drug-susceptible parasites.

Previous results have demonstrated that the acquisition of drug resistance could have an impact on parasite fitness, which could in turn influence other important biological properties involved in the regulation of proliferation and differentiation of parasites [6, 8]. As defined previously [6, 9], the fitness of *Leishmania* parasites can be measured by: i) capacity to survive, grow and generate infective metacyclic forms in the vector, ii) capacity to survive and grow in the mammalian host, and iii) capacity of transmission between the host and the vector.

Throughout its natural life cycle, *Leishmania* encounters adverse conditions that include: i) nutrient starvation and acidification of the medium, conditions that induce metacyclogenesis [10], ii) heat shock, when the parasite moves from growth at 28°C in the sandfly to 37°C inside the mammalian host macrophage, and iii) reactive oxygen species (ROS) and reactive nitrogen species (RNS), when it is phagocytized by macrophages of the host [11, 12]. *Leishmania* has evolved a broad spectrum of mechanisms to protect itself against these host defenses, including: i) enzymes that detoxify ROS and RNS [13], ii) use of thiols as antioxidant defenses [14], and iii) inhibition of the host's oxidative defense mechanisms [15].

However, it is not strictly true that all of the above increased the fitness capacity of all *L*. *donovani* resistant strains, as some strains showed little or no difference in their *in vivo* survival capacity compared to antimony-sensitive strains [16]. As suggested, other factors such as the genetic background of parasites could be important in the *in vivo* fitness capacity of *L*. *donovani* isolates that are clinically resistant to antimonials [16].

In this work, we evaluate whether *L*. *donovani* lines that are experimentally resistant to single anti-leishmanial drugs [amphotericin B (AmB), miltefosine (MIL), paromomycin (PMM) and trivalent antimony (Sb^III^)] and to drug combinations (AmB-MIL, AmB-PMM, AmB-Sb^III^, MIL-PMM and Sb^III^-PMM), present any advantages in their ability to bear the different stress conditions with respect to susceptible cells expressing the luciferase gene (Luc gene). For this purpose, we have studied the susceptibility of mixed promastigote populations under different stress conditions and their ability to infect and survive in mouse peritoneal macrophages. The results of this study using parasites that are experimentally resistant to single and multi-drug combinations are discussed in relation with their potential impact on future leishmaniasis control programs.

## Materials and Methods

### *L*. *donovani* culture conditions

*L*. *donovani* promastigotes (MHOM/ET/67/HU3) and the previously described derivative resistant lines A, M, P, S, AM, AP, AS, MP, and SP (resistant to AmB, MIL, PMM, Sb^III^, AmB+MIL, AmB+PMM, AmB+Sb^III^, MIL+PMM and Sb^III^+PMM, respectively) [4] were grown at 28°C in an RPMI 1640-modified medium (Invitrogen) supplemented with 20% heat-inactivated fetal bovine serum (hiFBS, Invitrogen). *L*. *donovani* with the luciferase gene integrated into the parasite genome (Luc line) was grown in the same conditions.

### Obtention of a bioluminescent *L*. *donovani* line

*Phothinus pyralis* luciferase gene (*luc*) was amplified from vector pX63NEO-3Luc [17] by PCR using the primers LucNcoIF 5’-GACGC**CCATGG**ATGGAAGACGCCAAAAACAT-3’ and LucNotIR 5’-GACGTA**GCGGCCGC**TTACAATTTGGACTTTCCGC-3’ including (in bold) *Nco*I and *Not*I restriction sites, respectively. The *luc* gene was then cloned into the *Nco*I*-Not*I sites of vector pLEXSY-hyg2 (Jena bioscience, Jena, Germany) which harbor a marker gene for selection with hygromycin-B (*hyg* gene). The vector generated was denominated pLEXSYHyg-Luc. In this construct, sequences of the 18S rRNA gene flanked the *luc* and *hyg* genes. Following linearization with *Swa*I, stationary promastigotes were transfected with 3 μg of linearized pLEXSYHyg-Luc plasmid to integrate the *luc* and *hyg* genes into the *18S rRNA* (*ssu*) locus by homologous recombination, using a previously described protocol [18]. Twenty-four hours after transfection, the culture medium was supplemented with 25 μg/mL of hygromycin-B. Hygromycin-resistant parasites were usually selected after 7 days. After establishing the transgenic parasites, they were plated onto 1.5% agar plates containing culture medium plus 100 μg/mL hygromycin-B. After 10 days incubating at 28°C, clones were selected on agar plates and further propagated in liquid RPMI-modified medium supplemented with 100 μg/mL hygromycin-B. Integration of the expression cassette into the *ssu* locus was confirmed by PCR using genomic DNA from the wild-type (WT) and transgenic strains of *L*. *donovani* (Luc line) as a template. For this purpose, we used primer pairs *ssu* forward primer F3001 5’-GATCTGGTTGATTCTGCCAGTAG-3’ and 5’ utr (*aprt*) reverse primer A1715 5’-TATTCGTTGTCAGATGGCGCAC-3’, and primer pairs *hyg* forward primer A3804 5’-CCGATGGCTGTGTAGAAGTACTCG-3’ and *ssu* reverse primers 3002 5’-CTGCAGGTTCACCTACAGCTAC-3’.

### Measurement of luciferase activity

Promastigotes and amastigotes isolated as described previously [19], were resuspended in HBS buffer (21 mM HEPES, 0.7 mM Na_2_HPO_4_, 137 mM NaCl, 5 mM KCl, and 6 mM D-glucose, pH 7.1) supplemented with 25 μM cell-permeable DMNPE-luciferin. After 15 minutes at room temperature, aliquots of this suspension (100 μL/well) were distributed into 96-well white polystyrene microplates. Luminescence was recorded with an Infinite F200 microplate reader (Tecan Austria GmbH, Austria). To measure the luciferase activity of intracellular amastigotes contained within infected cells, the Luciferase Assay System (Promega, Madison, Wis) was used according to the instructions of the manufacture. Luminescence was measured in the Infinite F200 microplate reader immediately after mixing.

### Determination of growth curve of mixed *L*. *donovani* lines

Log-phase promastigotes from the control (WT) and A, M, P, S, AM, AP, AS, MP and SP resistant lines were mixed in a 1:1 ratio with log-phase promastigotes from the Luc line (1x10^6^ mixed parasites/mL). Parasite density was microscopically determined every 24 h for a total of 144 h using Neubauer count chambers in order to monitor the growth. Parasite density in the WT line plus Luc line mixture was used as a control. Also, we evaluated the growth of each line in these mixed populations after 144 h of incubation, determining the luminescence as a measure of the cellular density of the Luc line. All growth experiments with the different parasite lines were performed in triplicate. In parallel, the mixed populations at a 1:1 ratio (4x10^6^ mixed parasites/mL) were sub-cultured every 48 h (logarithmic phase) and luminescence measured at the end of the first and second sub-culture.

### Proliferation of mixed *L*. *donovani* promastigote populations after different stress conditions

Promastigotes from the WT and resistant lines were mixed with the Luc line in a 1:1 ratio (4x10^6^ mixed parasites/mL), and the proliferation of resistant parasites exposed to different stress conditions (late stationary growth phase, starvation and heat shock plus pH modification) was compared to the Luc line by measuring the luminescence intensity. The mixture of the WT and Luc lines was used as a control for all experiments.

#### Late stationary phase growth profile

Promastigotes from resistant lines plus the Luc line or mixed WT plus Luc lines were cultured for 7 days and then sub-cultured for 48 h. Thereafter, an aliquot was used to determine the luminescence, and the remaining mixed population was kept for a further 5 days. A second sub-culture was performed and the luminescence determined after 48 h.

#### Starvation stress

We evaluated the proliferation capacity of mixed populations in starvation conditions by incubating parasites in a serum-free medium for 24 h. 20% hiFBS was then added to the medium and parasites were incubated for 48 h before determining the luminescence. The remaining mixed populations were subjected to the same stress once more, sub-cultured for 48 h in complete medium and the luminescence measured.

#### Heat shock and pH stresses

The mixed populations of parasites were incubated at 37°C for 24 h in an acidified culture medium, pH 5.4. The promastigotes were then diluted in culture medium, incubated for 48 h at 28°C and the luminescence was determined. Parasites were subjected to a second cycle of heat shock and pH stress before measuring the luminescence.

### Proliferation of mixed intracellular amastigote populations

For experiments studying intracellular amastigotes, mouse peritoneal macrophages were obtained as described previously [20] and plated at a density of 3 x 10^4^ or 3 x 10^5^ macrophages/well in 96-well white polystyrene microplates or 24-well tissue culture chamber slides, respectively, in an RPMI 1640 medium supplemented with 10% hiFBS, 2 mM glutamate, penicillin (100 U/mL) and streptomycin (100 μg/mL). Promastigotes from resistant or WT lines were mixed with the Luc line 1:1 ratio and maintained in culture for 6 days. Afterwards, the mixed populations of stationary phase cultures were used to infect macrophages at a macrophage/parasite ratio of 1:10. Six hours after infection at 35°C and 5% CO_2_, extracellular parasites were removed by washing with serum-free medium. Infected macrophages were maintained in culture medium at 37°C with 5% CO_2_ for 24 h and 96 h. To determine the infection index (% infection x amastigotes/macrophages), infected macrophages maintained in 24-well plates were fixed for 30 min at 4°C with 2.5% paraformaldehyde in PBS buffer (1.2 mM KH_2_PO_4_, 8.1 mM Na_2_HPO_4_, 130 mM NaCl, and 2.6 mM KCl, adjusted to pH 7), and permeabilized with 0.1% Triton X-100 in PBS for 30 min. Intracellular parasites and macrophages were detected by nuclear staining with ProLong Gold antifade reagent plus DAPI. To determine the intracellular proliferation profile of each line, infected macrophages maintained in 96-well plates were lysed and then the luminescence measured using the Luciferase Assay System (Promega).

### Animals

Eight-week-old male BALB/c mice were purchased from Charles River Breeding Laboratories and maintained in our Animal Facility Service under pathogen-free conditions. They were fed a typical rodent diet and given drinking water *ad libitum*. These mice were used to collect primary peritoneal macrophages.

### Ethics statement

All experiments were performed according to National/EU guidelines regarding the care and use of laboratory animals in research. Approval for these studies was obtained from the Ethics Committee of the Spanish National Research Council (CSIC, file CEA-213-1-11).

### Statistical analysis

Statistical comparisons between groups were performed using Student’s *t-*test. Differences were considered significant at a level of *p*<0.05.

## Results and Discussion

### Luciferase expression in promastigotes and intracellular amastigotes of *L*. *donovani*: A useful tool for measuring fitness

The firefly luciferase (*Luc*) [21] has proved to be a useful reporter gene for monitoring gene expression [22] and quantifying *Leishmania* infections in macrophages and animal models, with the overall aim of probing host-microbe interactions [23, 24]. To assess the feasibility of using bioluminescence as a quantitative indicator of parasite proliferation, studies were performed to correlate bioluminescence with parasite number. For this purpose, the *Luc* gene was amplified by PCR and cloned into pLEXSY-hyg2. The *LUC*-expressing vector was electroporated into *L*. *donovani* parasites which were then selected in the presence of hygromycin-B. To test whether luciferase activity correlated well with parasite number, 4-fold serial dilutions were prepared and their luciferase activity measured. An excellent linear correlation was observed between the number of transgenic promastigotes and the luminescence intensity (S1 Fig).

Transfectant parasites that overexpress luciferase (from now on, Luc line) were also tested for their ability to infect macrophages. Stationary-phase recombinant promastigotes were used to infect mouse peritoneal macrophages. Intracellular *Leishmania* infection was observed microscopically after DAPI staining and no significant differences were noted in the infectivity of the Luc line versus the WT line (S2A Fig). Furthermore, an excellent correlation was observed between amastigote numbers and luminescence intensity (S2B Fig). Collectively, these results strongly suggest that the Luc line constitutes a valuable tool for assessing the viability and dynamics of mixed populations.

### *Leishmania* growth profile of mixed populations

**Fig 1:**
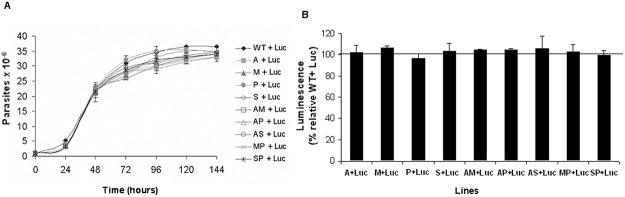
Growth profile of mixed promastigotes populations of *Leishmania* lines. (A) Log-phase promastigotes from WT and resistant lines were mixed at a 1:1 parasite ratio (1x10^6^ parasites/ml) with log-phase promastigotes from Luc line (Luc), and the number of parasites were monitored every 24 h. (B) Luminescence of 10^7^ promastigotes from mixed populations was determined after 6 days of incubation in standard culture medium. Results are the average of three independent experiments ± SD. Horizontal line indicates the threshold at which luminescence levels have been modified with respect to control (WT+Luc).

The promastigote number was evaluated every day for 6 days so that the growth features of each resistant line, or the WT line mixed in a 1:1 parasite ratio with the Luc line, could be studied and compared. We found that all mixed populations showed a similar growth profile as the control (WT+Luc) (Fig 1A). Moreover, the luminescence values were similar for each of these mixed populations after the 6^th^ day of culture (Fig 1B). These results clearly indicate that the Luc line was present in the same ratio in all mixed populations and, therefore, there was no predominance of one line over another line under these conditions.

### Proliferation of mixed *Leishmania* populations after exposure to different stress conditions

To evaluate whether there was predominance of any resistant lines over the susceptible Luc line, promastigotes from resistant lines and the Luc line were mixed in a 1:1 parasite ratio and grown without stress or exposure to different stresses. To assess the growth recovery, the luminescence intensity of mixed populations was determined in all cases after 48 h of culture in standard conditions. The WT plus Luc lines (WT+Luc) mixture was used as a control. The total number of mixed parasite cultures shows no significant differences between WT+Luc and the resistant lines+Luc in the different stress conditions, ranging indistinctly between 22-32x10^7^ mixed parasites/mL.

#### Without nutritional stress

Mixed populations were sub-cultured every 48 h to maintain continuous logarithmic growth and adequate nutritional requirements. After two sub-cultures, luminescence analysis did not reveal any significant differences between mixed populations in relation to controls (ranging from 90 to 118% indistinctly). Therefore, in the absence of nutritional stress, none of the *Leishmania* lines showed growth advantage relative to the others.

#### Late stationary phase assays

**Fig 2:**
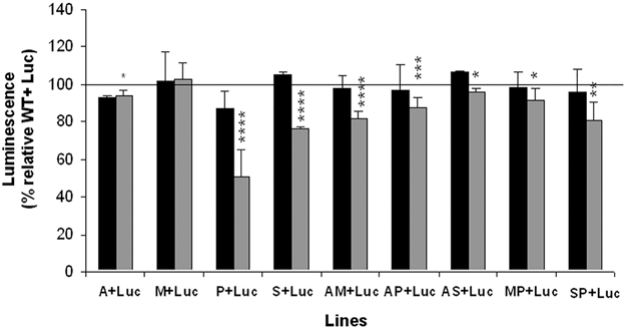
*Leishmania* resistant lines are more tolerant to stress in the late stationary growth phase. Promastigotes from WT and resistant lines were mixed at a 1:1 parasite ratio with promastigotes from Luc line (Luc), maintained in standard culture medium for 7 days and the luminescence of 10^7^ promastigotes was determined at 48 h of the first (black columns) and second (gray columns) sub-culture cycle. Results are the average of four independent experiments ± SD. Statistically differences using Student's *t* test are indicated relative to the control values (*, *p*<0.05; **, *p*<0.01; ***, *p*<0.005; ****, *p*<0.001). Horizontal line indicates the threshold at which luminescence levels have been modified with respect to control (WT+Luc).

Major factors determining entry into the stationary phase are pH and the availability of nutrients. Parasite multiplication during culture causes consumption of nutrients resulting in nutrient deficiency and medium acidification, conditions that are known to induce metacyclogenesis [10]. To assess the response during the late stationary phase after two successive culture cycles, mixed populations were maintained for 7 days, sub-cultured and then the luminescence determined. All mixed populations exhibited a similar reaction to stress at the first sub-culture, showing a similar luminescence value (Fig 2, black columns). However, in the second sub-culture event, a significant decrease in luminescence with respect to the control was observed for all mixed populations, except for that containing the M line (Fig 2, gray columns). The lines that showed a higher ratio of growth were P, S and AM, since the mixed populations P+Luc, S+Luc, and AM+Luc had luminescence values of 51%, 76% and 82%, respectively, versus the 100% luminescence of the mixture WT+Luc (Fig 2, horizontal line). These results suggested that the resistant lines are more tolerant to stress conditions present in the late stationary phase than the Luc line, because their higher growth rates are responsible for the displacement mentioned above. Also note that the AM resistant line has an increased growth rate compared to the single-drug resistant lines A and M (Fig 2, gray columns). The different growth profiles observed could be due to the metabolic adaptations which all converge on the higher tolerance of the resistant lines to stress conditions, as recently described [25].

#### Starvation

**Fig 3:**
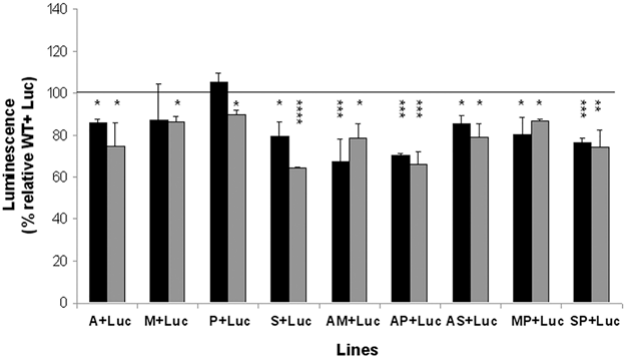
*Leishmania* resistant lines show higher survival in starvation. Promastigotes from WT and resistant *Leishmania* lines were mixed at a 1:1 parasite ratio with promastigotes from Luc line (Luc), incubated in serum-free medium for 24 h, supplemented with 20% hiFBS and the luminescence of 10^7^ promastigotes was determined at 48 h of the first (black columns) and second (gray columns) starvation cycle. Results are the average of four independent experiments ± SD. Statistically differences using Student's *t* test are showed relative to the control values (*, *p*<0.05; **, *p*<0.01; ***, *p*<0.005; ****, *p*<0.001). Horizontal line indicates the threshold at which luminescence levels have been modified with respect to control (WT+Luc).

We assessed the proliferation capacity of mixed *Leishmania* populations under starvation conditions. This was to determine whether the predominance of resistant lines observed during the stationary phase was only due to a difference in starvation stress tolerance or due to all of the stresses that occur during the stationary phase of growth. For this purpose, we incubated mixed populations in serum-free medium for 24 h. Then we monitored the luminescence in complete media and the remaining mixed populations were subjected to the same stress once more before the analysis. After the first cycle, all resistant lines, except M and P lines, predominate over the susceptible Luc line (Fig 3, black columns), with significantly higher differences existing after the second starvation cycle (Fig 3, gray columns). S and AP *Leishmania* lines showed a marked predominance over the Luc line, since the S+Luc and AP+Luc populations exhibited luminescence values relative to the control (WT+Luc) of 64% and 66%, respectively (Fig 3, horizontal line). However, the comparison of single- and dual-drug resistant lines indicated a greater advantage for all dual resistant lines (except for the AS line) when compared to any of their respective single-drug resistant lines (Fig 3). These results suggest that, in the absence of nutrients, the resistant lines, and more specifically those resistant to drug combinations, present higher survival rates than the susceptible Luc line since they are able to adapt their energy metabolism to better cope with starvation, presumably by increasing their tolerance to this stress.

#### Heat shock plus pH stress

**Fig 4:**
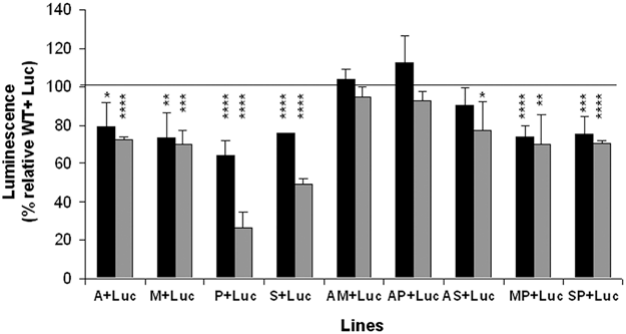
*Leishmania* resistant lines are more tolerant to heat shock and pH stress. Mixed promastigotes from WT and resistant lines at a 1:1 parasite ratio were incubated in culture medium, pH 5.4 at 37 ºC for 24 h. Then, mixed populations were incubated at 28 ºC and pH 7.2 and luminescence of 10^7^ promastigotes was evaluated at the first (black columns) and second (gray columns) stress cycle. Results are the average of four independent experiments ± SD. Statistically differences using Student's *t* test are showed relative to the control values (*, *p*<0.05; **, *p*<0.01; ***, *p*<0.005; ****, *p*<0.001). Horizontal line indicates the threshold at which luminescence levels have been modified with respect to control (WT+Luc).

The survival capacity of promastigotes is not only determined by their nutrient needs, but also by their capacity to resist stresses encountered inside the macrophages, such as acidic pH and high temperatures. In this experiment mixed populations were exposed to two culture cycles at 37°C and pH 5.4 for 24 h and luminescence determined in standard culture conditions. Under these stress conditions, we observed that all the resistant lines predominated over the Luc line at both cycles (Fig 4, black and gray columns respectively), except the AM and AP lines, and the AS line which only predominated over the Luc line after the second cycle (Fig 4, gray columns). This observation could be associated with the fact that resistance to drug combinations AmB plus other anti-leishmania drugs (MIL, PMM and Sb^III^) confer metabolic changes associated with the loss of this advantage. This interesting observation will be considered in future studies to gain more inside about the possible effect of AmB in the resistant phenotype of drug combinations. Luminescence values with respect to the control (WT+Luc) for mixed populations ranged from 27 to 77%, for P+Luc to AS+Luc populations, respectively. Furthermore, none of the dual-drug resistant lines exhibited a higher fitness than their respective single-drug resistant lines (Fig 4). These results suggest that resistant lines are more tolerant to heat shock and pH stress conditions than the Luc line, which could facilitate a higher survival rate inside the macrophage and an increased capacity to deal with the natural stresses that the parasites face throughout their life cycle.

### Proliferation of mixed intracellular amastigotes populations

**Fig 5:**
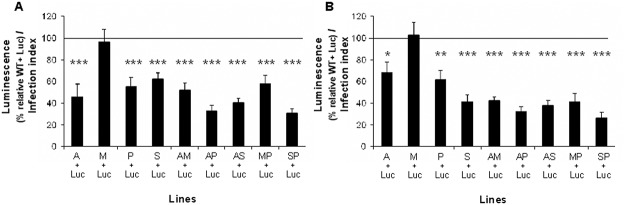
Intracellular amastigotes of *Leishmania* resistant lines present higher capacity to survive in mouse peritoneal macrophages. Mouse peritoneal macrophages were plated in 96-well white polystyrene microplates and infected during 6 h with promastigotes of 6 days of culture from resistant or WT lines mixed with Luc line. Infected macrophages were maintained in culture medium at 37°C with 5% CO_2_ during 24 h (A) or 96 h (B) and luminescence was determined and normalized by infection index. Results are the average of three independent experiments ± SD. Statistically differences using Student's *t* test are showed relative to the control values (*, *p*<0.05; **, *p*<0.01; ***,*p*<0.001). Horizontal line indicates the threshold at which luminescence levels have been modified with respect to control (WT+Luc).

Within the mammalian host, *Leishmania* promastigotes differentiate into amastigotes and multiply predominantly inside macrophages, where they are exposed to stress, including starvation, acidic pH, high temperatures (heat shock) and ROS and RNS production [11, 26]. To determine whether intracellular amastigotes from resistant lines were able to displace *in vitro* intracellular amastigotes from the susceptible Luc line, macrophages were infected with mixed populations of promastigotes taken from 6 day-old cultures where, as shown in Fig 1, no differences were observed in proliferation and the Luc line ratio. The infection index and luminescence of intracellular amastigotes were determined at 24 and 96 h post-infection to assess their infectivity and survival rates in mouse peritoneal macrophages. The infection indexes were similar in all cases, with values ranging for 24 h between 183±22 and 234±28, and for 96 h between 182±27 and 250±34. The results showed that in the early stage of macrophage infection (24 h) all the resistant lines, except the M line, had a significant predominance over the Luc line compared to the control (Fig 5A). The different lines showed a range of luminescence between 31 and 63% from SP+Luc and S+Luc populations, respectively, compared to the control (Fig 5A). These results could be due to: i) a higher percentage of metacyclic parasites, ii) tolerance to oxidative stress, and/or iii) tolerance to acidic pH and high temperatures of the resistant lines compared to the susceptibility of the Luc line. Also, in the late stage of infection (96 h), the resistant lines, again with the exception of the M line, were able to fully benefit from their initial advantage (Fig 5B). They showed a range of luminescence between 27 and 68% from SP+Luc and A+Luc populations, respectively, compared to the luminescence produced by the control (Fig 5B). Additionally, there were no significant differences on predominance of resistant lines between 24 and 96 h, with the exception of the S line (*p*<0.05). These results suggest that the predominance of some resistant lines over the Luc line is the result of a higher infection rate during the initial stage which reflects as a higher survival rate of intracellular amastigotes within macrophages.

*Leishmania* have successfully adapted to different environments for thousands of years and developed a highly flexible nature. Their survival capacity mainly relies on their ability to suppress oxidative outbursts of the host defense mechanism [15] and on a unique oxidant-protective redox metabolism, where thiols play a key role in antioxidant defenses [14]. In this regard, we have previously described that the resistant lines, except the M and S lines, had higher non-protein thiol levels than the WT line [4], which could contribute to a greater parasite survival rate within the host macrophages.

It has been demonstrated that some *L*. *donovani* strains resistant to antimonials have a more variable and markedly higher capacity of *in vivo* infection compared to antimony susceptible *Leishmania* strains [16]. Strains resistant to antimony also have a higher metacyclogenic capacity [27] and have specifically evolved extra mechanisms to manipulate their host cells in order to avoid antimony-induced stress [28]. Such adaptations would not only improve the parasites survival capacity when stressed by antimony, but would also favor their survival in drug-free conditions.

Since Sb^III^, AmB and MIL kill *Leishmania* through a common cell death pathway to achieve apoptosis, strains resistant to one or more of these drugs could develop tolerance to apoptosis, which would grant them a higher survival rate in macrophages, as we have observed with our *Leishmania* resistant lines (Fig 5B).

In conclusion, the experiments using our transgenic *Leishmania* luc line have clearly demonstrated and validated the fact that *Leishmania* lines experimentally resistant to individual and combinatorial anti-leishmanial drugs have an increased fitness compared to *Leishmania* susceptible lines, probably as a consequence of their metabolic adaptations which all converge on the higher tolerance to stress conditions, as recently described [25]. Subsequently, they also have a better chance of survival. However, although this approach using promastigotes for assessing the viability and dynamics of mixed populations have important advantages, their use on intracellular amastigotes has some methodological limitations. Therefore, the emergence and spread of drug-resistant parasites in the field will probably result in a greater competitive fitness cost with respect to susceptible parasites, plus negative effects on the chemotherapy strategies used to control leishmaniasis.

## Supporting Information

S1 Fig Correlation between luciferase activity and number of promastigotes. 4-fold serial dilutions were prepared and parasites counted microscopically using Neubauer count chambers. Luminescence intensity was recorded in a.u. (arbitrary units) using an Infinite F200 microplate reader. These results represent three independent experiments.
(TIF)

S2 Fig Infection index and correlation between luciferase activity and number of amastigotes. (A) Intracellular *Leishmania* infection of WT and Luc lines was observed microscopically after DAPI staining. Results are the mean ± S.D. of three independent experiments. (B) 2-fold serial dilutions were prepared and parasites counted microscopically using Neubauer count chambers. Luminescence intensity expressed as a.u. (arbitrary units) was measured using an Infinite F200 microplate reader. Results are representative of three independent experiments.
(TIF)

## References

[1] Alvar J, Vélez ID, Bern C, Herrero M, Desjeux P, Cano J, et al WHO Leishmaniasis control team. Leishmaniasis worldwide and global estimates of its incidence. PLoS One 2012;7: e356717. 10.1371/journal.pone.0035671 PMC3365071 22693548

[2] Sundar S. Drug resistance in Indian visceral leishmaniasis. Trop Med Int Health 2001;6: 849–854. 11703838 10.1046/j.1365-3156.2001.00778.x

[3] Olliaro PL. Drug combinations for visceral leishmaniasis. Curr Opin Infect Dis. 2010;23: 595–602. 10.1097/QCO.0b013e32833fca9d 20871400

[4] Garcia-Hernandez R, Manzano JI, Castanys S, Gamarro G. *Leishmania donovani* develops resistance to drug combinations. PLoS Negl Trop Dis. 2012;6: e1974 10.1371/journal.pntd.0001974 23285310 PMC3527373

[5] Sundar S, More DK, Singh MK, Singh VP, Sharma S, Makharia A, et al Failure of pentavalent antimony in visceral leishmaniasis in India: report from the center of the Indian epidemic. Clin Infect Dis. 2000;31: 1104–1107. 11049798 10.1086/318121

[6] Vanaerschot M, Huijben S, Van den Broeck F, Dujardin JC. Drug resistance in vector borne parasites: multiple actors and scenarios for an evolutionary arms race. FEMS Microbiol Rev. 2014;38: 41–55. 10.1111/1574-6976.12032 23815683

[7] Ait-Oudhia K, Gazanion E, Oury B, Vergnes B, Sereno D. The fitness of antimony-resistant *Leishmania* parasites: lessons from the field. Trends Parasitol. 2011;27: 141–142. 10.1016/j.pt.2010.12.003 21216196

[8] Sereno D, Guilvard E, Maquaire S, Cavaleyra M, Holzmuller P, et al Experimental studies on the evolution of antimony-resistant phenotype during the in vitro life cycle of *Leishmania infantum*: implications for the spread of chemoresistance in endemic areas. Acta Trop. 2001;80: 195–205. 11700176 10.1016/s0001-706x(01)00154-1

[9] Vanaerschot M, Decuypere S, Berg M, Roy S, Dujardin JC. Drug-resistant microorganisms with a higher fitness—can medicines boost pathogens?. Crit Rev Microbiol. 2012;39: 384–394. 10.3109/1040841X.2012.716818 22950457

[10] Zilberstein D, Shapira M. The role of pH and temperature in the development of *Leishmania* parasites. Annu Rev Microbiol. 1994;48: 449–470. 7826014 10.1146/annurev.mi.48.100194.002313

[11] Wilson ME, Jeronimo SMB, Pearson RD. Immunopathogenesis of infection with the visceralizing *Leishmania* species. Microb Pathog. 2005;38: 147–160. 15797810 10.1016/j.micpath.2004.11.002

[12] Murray HW, Nathan CF. Macrophage microbicidal mechanisms in vivo: reactive nitrogen versus oxygen intermediates in the killing of intracellular visceral *Leishmania donovani* . J Exp Med. 1999;189: 741–746. 9989990 10.1084/jem.189.4.741 PMC2192937

[13] Castro H, Tomas AM. Peroxidases of trypanosomatids. Antioxid Redox Signal 2008;10: 1593–1606. 10.1089/ars.2008.2050 18498224

[14] Mehlotra RK. Antioxidant defense mechanisms in parasitic protozoa. Crit Rev Microbiol. 1996;22: 295–314. 8989515 10.3109/10408419609105484

[15] Bhardwaj S, Srivastava N, Sudan R, Saha B. *Leishmania* interferes with host cell signaling to devise a survival strategy. J Biomed Biotechnol, 2010; 2010: 109189–109202. 10.1155/2010/109189 20396387 PMC2852600

[16] Vanaerschot M, De Doncker S, Rijal S, Maes L, Dujardin JC, Decuypere S. Antimonial resistance in *Leishmania donovani* is associated with increased in vivo parasite burden. PLoS One 2011;6: e23120 10.1371/journal.pone.0023120 21829701 PMC3148249

[17] Luque-Ortega JR, Rivero-Lezcano OM, Croft SL, Rivas L. In vivo monitoring of intracellular ATP levels in *Leishmania donovani* promastigotes as a rapid method to screen drugs targeting bioenergetic metabolism. Antimicrob Agents Chemother. 2001;45: 1121–1125. 11257025 10.1128/AAC.45.4.1121-1125.2001 PMC90434

[18] Pérez-Victoria FJ, Sánchez-Cañete MP, Castanys S, Gamarro F. Phospholipid translocation and miltefosine potency require both *L*. *donovani* miltefosine transporter and the new protein LdRos3 in Leishmania parasites. J Biol Chem. 2006;28: 23766–23775. 10.1074/jbc.M605214200 16785229

[19] Rochette A, Raymond F, Ubeda JM, Smith M, Messier N, Boisvert S, et al Genome-wide gene expression profiling analysis of *Leishmania major* and *Leishmania infantum* developmental stages reveals substantial differences between the two species. BMC Genomics 2008; 9: 255 10.1186/1471-2164-9-255 18510761 PMC2453527

[20] Seifert K, Pérez-Victoria FJ, Stettler M, Sánchez-Cañete MP, Castanys S, Gamarro F, et al Inactivation of the miltefosine transporter, LdMT, causes miltefosine resistance that is conferred to the amastigote stage of *Leishmania donovani* and persists in vivo. Int J Antimicrob Agents 2007;30: 229–235. 17628445 10.1016/j.ijantimicag.2007.05.007

[21] Gould SJ, Subramani S. Firefly luciferase as a tool in molecular and cell biology. Anal Biochem.1988;175: 5–13. 3072883 10.1016/0003-2697(88)90353-3

[22] Welsh S, Kay SA. Reporter gene expression for monitoring gene transfer. Curr Opin Biotechnol.1997;8: 617–622. 9353237 10.1016/s0958-1669(97)80038-9

[23] Roy G, Dumas C, Sereno D, Wu Y, Singh AK, Tremblay MJ, et al Episomal and stable expression of the luciferase reporter gene for quantifying *Leishmania spp*. infections in macrophages and in animal models. Mol Biochem Parasitol. 2000;110: 195–206. 11071276 10.1016/s0166-6851(00)00270-x

[24] Lang T, Goyard S, Lebastard M, Milon G. Bioluminescent *Leishmania* expressing luciferase for rapid and high throughput screening of drugs acting on amastigote-harbouring macrophages and for quantitative real-time monitoring of parasitism features in living mice. Cell Microbiol. 2005;7: 383–392. 15679841 10.1111/j.1462-5822.2004.00468.x

[25] Berg M, García-Hernández R, Cuypers B, Vanaerschot M, Manzano JI, Poveda JA, Ferragut JA, Castanys S, Dujardin JC, Gamarro F. Experimental resistance to drug combinations in *Leishmania donovani*: metabolic and phenotypic adaptations. Antimicrob Agents Chemother. 2015; 10.1128/AAC.04231-14 PMC4356759 25645828

[26] Wei XQ, Charles IG, Smith A, Ure J, Feng CJ, Huang FP, et al Altered immune-responses in mice lacking inducible nitric-oxide synthase. Nature 1995;375: 408–411. 7539113 10.1038/375408a0

[27] Ouakad M, Vanaerschot M, Rijal S, Sundar S, Speybroeck N, Kestens L, et al Increased metacyclogenesis of antimony-resistant *Leishmania donovani* clinical lines. Parasitol. 2011;138: 1392–1399. 10.1017/S0031182011001120 21819638

[28] Haldar AK, Yadav V, Singhal E, Bisht KK, Singh A, Bhaumik S, et al *Leishmania donovani* isolates with antimony-resistant but not-sensitive phenotype inhibit sodium antimony gluconate-induced dendritic cell activation. PLoS Pathog. 2010;6: e1000907 10.1371/journal.ppat.1000907 20502630 PMC2873921

