# Supplementary figures and images for: Fitness of Leishmania donovani Parasites Resistant to Drug Combinations
Source: PLoS Negl Trop Dis. 2015 Apr 7;9(4):e0003704. doi: 10.1371/journal.pntd.0003704 (PMC4388546; doi:10.1371/journal.pntd.0003704)

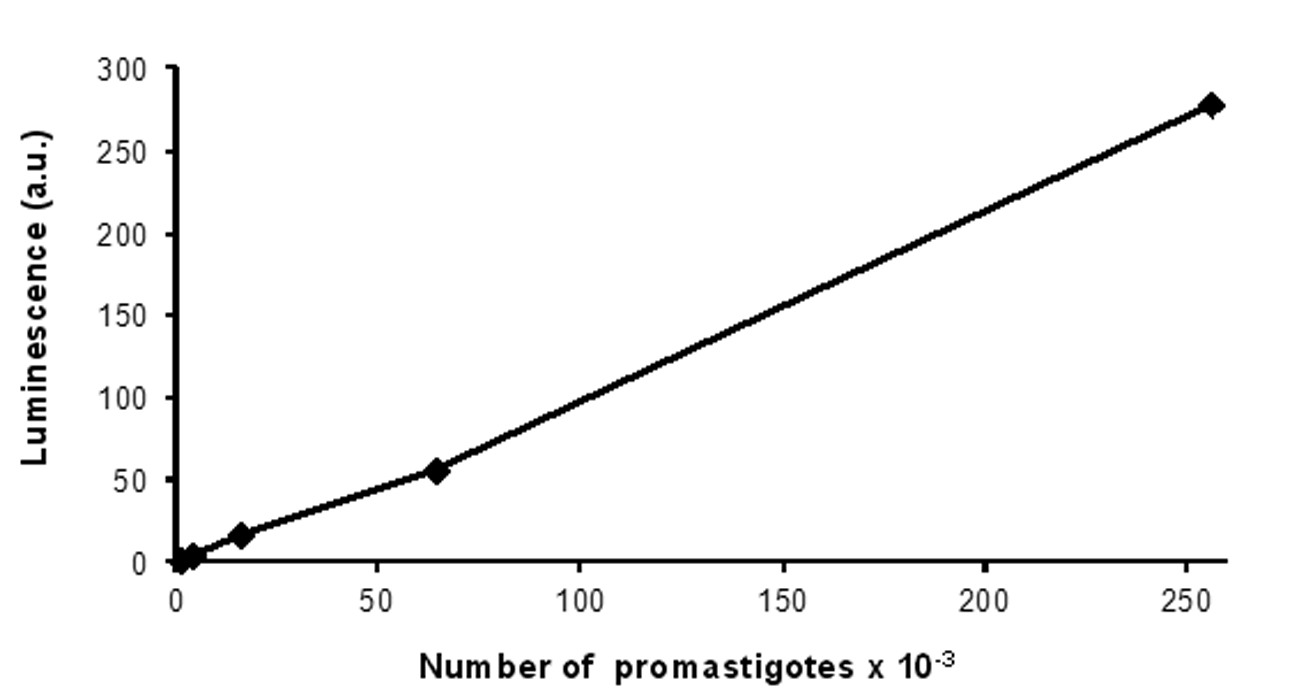

Supplement: S1 Fig — 4-fold serial dilutions were prepared and parasites counted microscopically using Neubauer count chambers. Luminescence intensity was recorded in a.u. (arbitrary units) using an Infinite F200 microplate reader. These results represent three independent experiments. (TIF) [file pntd.0003704.s001.tif]

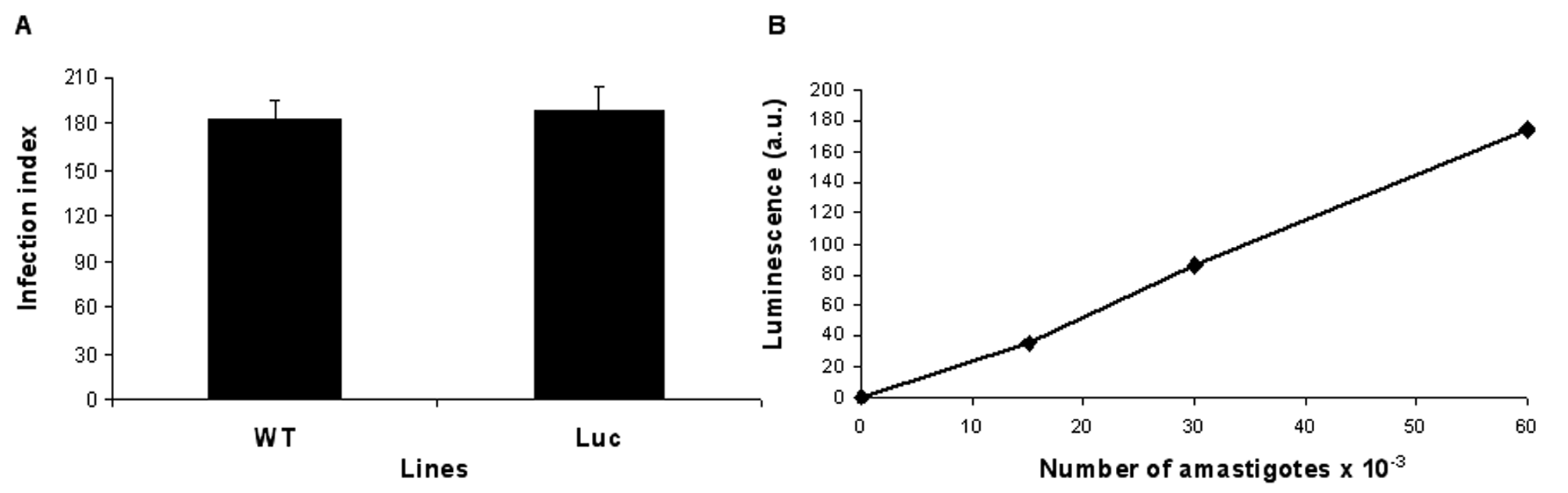

Supplement: S2 Fig — (A) Intracellular Leishmania infection of WT and Luc lines was observed microscopically after DAPI staining. Results are the mean ± S.D. of three independent experiments. (B) 2-fold serial dilutions were prepared and parasites counted microscopically using Neubauer count chambers. Luminescence intensity expressed as a.u. (arbitrary units) was measured using an Infinite F200 microplate reader. Results are representative of three independent experiments. (TIF) [file pntd.0003704.s002.tif]
